# E-Liquid Flavor Preferences and Individual Factors Related to Vaping: A Survey among Dutch Never-Users, Smokers, Dual Users, and Exclusive Vapers

**DOI:** 10.3390/ijerph16234661

**Published:** 2019-11-22

**Authors:** Kim AGJ Romijnders, Erna JZ Krüsemann, Sanne Boesveldt, Kees de Graaf, Hein de Vries, Reinskje Talhout

**Affiliations:** 1 Centre for Health Protection, National Institute for Public Health and the Environment (RIVM), Antonie van Leeuwenhoeklaan 9, 3721 MA Bilthoven, The Netherlandsreinskje.talhout@rivm.nl (R.T.); 2 Department of Health Promotion, School for Public Health and Primary Care (CAPHRI), Maastricht University, Universiteitssingel 40, 6229 ER Maastricht, The Netherlands; hein.devries@maastrichtuniversity.nl; 3 Division of Human Nutrition and Health, Wageningen University, Stippeneng 4, 6708 WE Wageningen, The Netherlands; sanne.boesveldt@wur.nl (S.B.); kees.degraaf@wur.nl (K.d.G.)

**Keywords:** electronic cigarettes, flavors, preference, smoking, vaping, knowledge, attitude, deliberation, perceived susceptibility

## Abstract

Appealing product characteristics, such as flavors, may stimulate e-cigarette use. While switching to e-cigarettes may reduce harm for smokers, concerns exist about e-cigarette use among never-smokers. The role of flavors in the decision to switch to or refrain from vaping is unclear. This study used a bottom–up approach to investigate the relation between flavor preferences and individual factors related to vaping between various user groups. A cross-sectional survey was conducted among never-users (*n* = 407), smokers (*n* = 138), dual users (*n* = 122), and exclusive vapers (*n* = 61) in the Netherlands. Demographics, attractiveness of product characteristics, flavor preferences, and individual factors related to vaping (knowledge, trust, perceived susceptibility, attitude, social influence, deliberation, and intention) were assessed. The availability of different flavors was the most attractive characteristic of e-cigarettes. Dual users and exclusive vapers had most often used tobacco and menthol/mint flavors when they first started vaping. Compared to dual users, exclusive vapers currently used more fruit and sweet flavors. Never-users who were interested in trying an e-liquid flavor had more knowledge about and a more positive attitude towards e-cigarettes. Smokers who were interested in trying a flavor had a more positive attitude towards e-cigarettes and experienced the social influence towards not using e-cigarettes as less strong than those who did not want to try any flavor. Hence, individual factors related to vaping differed depending on whether never-users and smokers wanted to try an e-liquid flavor. This means that flavors may moderate differences found in individual factors related to vaping, or vice versa.

## 1. Introduction

Although vaping prevalence in the Netherlands is currently rather low (3%) [1], the use of electronic cigarettes (e-cigarettes) increased worldwide in recent years [2,3]. The majority of e-cigarette users are former or current smokers [4,5,6] and literature has showed that e-cigarette use (i.e., vaping) may be associated with smoking cessation and reduction [7,8]. In the Netherlands, e-cigarettes are more often used by daily smokers (12%) compared to non-daily smokers (4%). Worldwide, vaping is also becoming increasingly popular among adolescent non- and never-smokers [3,9,10]. The regulation of e-cigarettes in order to optimize public health benefits is challenging and is currently an important topic of debate. Compared to cigarette smoking, vaping may reduce harm among smokers [11,12,13,14], but literature showed that e-cigarettes contain toxic ingredients [11]. In addition, concerns have been raised that vaping may contribute to nicotine addiction and the renormalization of cigarette smoking in adolescent never-smokers [3,15,16,17,18]. Consequently, from a public health perspective, the initiation of vaping by current non- and never-smokers and, thereby, exposure to potentially toxic ingredients should be prevented [18].

Research is needed to better understand differences between the initiation of e-cigarette use by current smokers versus non-smokers in order to inform regulators about policy making regarding e-cigarettes in order to develop targeted health communication for smokers, non-smokers, and e-cigarette users. Previous studies found differences in individual factors related to e-cigarettes among never-users, smokers, dual users, and e-cigarette users [19]. Individual factors that were found to differ included, for example, knowledge, perceived susceptibility, severity, trust, attitudes, deliberation, social influence, and intention [19,20,21,22,23]. Furthermore, literature showed that the importance of product characteristics such as design, price, and flavors may differ between adult smokers and adolescent non-smokers [24,25,26,27]. However, the relation between e-cigarette product characteristics and individual-level factors has been neglected.

A recent study hypothesized that there is an important interplay between individual-level factors and the characteristics of tobacco products [28]. Since product characteristics (e.g., flavors, design, and price) influence e-cigarette appeal [24,25,26,27] and may influence a person’s attitude towards e-cigarettes [24,28], such an interplay may also exist for e-cigarettes. However, thus far, most studies on e-cigarettes focused either on product characteristics [29], or on socio-cognitive factors related to vaping behavior [30]. In contrast, researchers in the food and nutrition domain have already recognized the importance of the interaction between product characteristics and individual-level decision-making factors in food choice [31,32,33]. For example, a model by Shepherd [31] shows that food choice is influenced by the interaction between physical or chemical properties of food, such as flavors (product factor), and the individual’s perception of and attitude towards those sensory properties (individual-level factors). Furthermore, flavors and other sensory properties are recognized as by far the most important factors in the acceptance and rejection of food products [32].

Similarly, since e-liquid flavors are recognized as an important reason for e-cigarette use [24], flavors may interact with individual-level factors related to vaping. Moreover, the availability of many different, mostly sweet, e-liquid flavors is an important reason for vaping among different types of users [24,29]. Research showed that for most e-cigarette users, and in particular for youth, the first and current e-liquid had a flavor other than tobacco [26,34,35]. In addition, flavors increase the probability of choosing e-cigarettes in an online discrete choice experiment among youth, for both never-users and ever-users of e-cigarettes [36]. Therefore, besides investigating product characteristics as reasons for e-cigarette use, additional research is needed to investigate the interaction between flavors as an e-cigarette product characteristic and individual factors related to vaping.

To increase our understanding of differences in e-cigarette appeal between user groups, this study firstly investigates which product characteristics are found attractive by Dutch never-users, smokers, dual users, and exclusive vapers. Secondly, we aim to determine the flavor preferences of Dutch never-users, smokers, dual users, and exclusive vapers. Thirdly, we aim to explore whether eight individual factors related to vaping differ between never-users and smokers who reported to be interested in trying an e-liquid flavor compared to those who reported not to be interested in trying any e-liquid flavor.

## 2. Materials and Methods

A cross-sectional survey was conducted in the Netherlands among never-users of e-cigarettes and cigarettes, cigarette smokers, dual users of e-cigarettes and cigarettes, and e-cigarette users. The survey was administered in May 2017 through the online survey panel Flycatcher, which is an ISO-certified independent research panel specialized in online research [37]. The study was approved by the Medical Ethics Committee of Zuyderland – Zuyd (17-N-88). The recruitment, participant characteristics, and survey were previously described in Romijnders, et al. [38].

### 2.1. Recruitment and Participant Characteristics

In total, 12,750 invitations were sent to panelists who met the following inclusion criteria: being able to understand Dutch; being aware of e-cigarettes; aged 13–17 years (adolescents) or 18 years and older (adults). Sample size was determined based on a power of 80% to identify a minimal difference of 1 on a 7-point Likert scale for attitude (based on previous literature [39]) as significant at *p* < 0.05. The sample cannot be considered representative of the Dutch population, as oversampling for the smokers and e-cigarette users was performed in order to achieve sufficient observations. Participants were asked to provide consent before the start of the survey. Parents of panelists under the age of 18 had previously provided consent for participation of their child in research questionnaires. Overall, 1307 surveys were completed, and the response rate was 10.3% (69.7% for adults, *n* = 694; 5.2% for adolescents, *n* = 613). For the current study, respondents were eligible if they met the definition of one of the following user groups (see Supplementary file 1 for the survey items used [38]): never-users are participants who reported to never have smoked and never used an e-cigarette; smokers are participants who reported to currently exclusively use cigarettes on a daily or weekly basis; dual users are participants who reported to currently simultaneously use cigarettes on a daily or weekly basis and e-cigarettes on a daily or weekly basis; vapers are participants who reported to currently exclusively use e-cigarettes on a daily or weekly basis [40].

It should be noted that these definitions, similar to studies performed before [39], includes individuals who had no history of smoking prior to becoming an exclusive vaper. In addition, and due to the cross-sectional nature of the data, no transitory phases for dual users and exclusive vapers can be determined [40]. We aimed for mutually exclusive groups. Hence, as the group of exclusive vapers may also include former smokers and the group of exclusive smokers may also include former vapers, former users were not included as a separate group. An overview of the items used to determine whether respondents met our definitions can be found in Supplementary file 1 [38]. In total, 728 participants met the eligibility criteria of this study. Of those, 394 were adults (62.4% female) and 334 were adolescents (46.7% female).

### 2.2. Survey

The current study included measures on basic demographics, attractiveness of e-cigarettes, flavor preferences, and individual factors related to vaping. The survey included routing to ensure that participants were asked about relevant items only (e.g., never-users were not asked which flavor their first e-cigarette had). A full overview of the items and concepts is available in Supplementary file 1 [38].

First, participants were asked about basic demographics, and smoking and vaping characteristics [19,24,40,41,42,43,44]. Educational level was determined based on the Dutch version of the International Standard Classification of Education (ISCED) [45].

Second, participants were asked to evaluate the attractiveness of eight product characteristics of e-cigarettes from a predetermined list using a check all that apply (CATA) approach that was based on previous research [19,24]. For the items that were used to assess demographics and attractiveness of product characteristics, see Supplementary file 1 [38].

Third, interest in trying an e-liquid flavor (for never-users and smokers), and the first and current e-liquid flavors used (for dual and e-cigarette users) were assessed. For the items that were used to assess flavor preferences, see Supplementary file 1 [38]. To assess flavor interest among never-users and smokers, multiple flavor categories [46] (CATA) or the option “none of the flavors” were selected. If the latter answer option was selected, no flavor category could be selected simultaneously. E-cigarette users and dual users were asked about their current and first e-liquid flavor used: “Which flavor do you currently use most? If possible, please specify the name of the flavor” and “Which flavor did you try first? If possible, please specify the name of the flavor.” For both current and first flavor used, dual users and e-cigarette users could select only one flavor category [46] and had to specify their choice through an open question. The answer options for never-users, smokers, dual users and e-cigarette users were: tobacco, menthol/mint, nuts, herbs, spices, coffee/tea, cocktails, alcohol, other, sodas, sweet (chocolate, vanilla, desserts, or other), fruit, milk products, candy, floral, unflavored, and none of the flavors. The closed answer options that were used to assess flavor preference in all user groups were recoded according to the thirteen main categories of the recently published e-liquid flavor wheel [47], except for the option “none of the flavors”. Recoding the reported flavor preferences resulted in the in the following thirteen main categories: tobacco (survey item: tobacco), menthol/mint (survey item: menthol/mint), nuts (survey item: nuts), spices (survey items: herbs, spices), coffee/tea (survey items: coffee; tea), alcohol (survey items: alcohol, cocktail; alcohol, other), other beverages (survey items: soda; sweet, other), fruit (survey item: fruit), dessert (survey items: sweet, dessert; milk product), other sweets (survey items: sweet, chocolate; sweet, vanilla), candy (survey items: sweet, candy), other flavors (survey items: floral; other) and unflavored (survey item: unflavored). Open answers from dual and e-cigarette users were assessed by two authors (KR and EK) to support recoding of the closed answers according to the categories of the e-liquid flavor wheel [47]. In some cases, multiple survey items were associated with one flavor category (e.g., the survey answer options “sweet, chocolate” and “sweet, vanilla” were both recoded to the other sweets flavor category). For equal weight of the flavor categories, each participant could obtain a maximum score of “1” for each flavor category. Thus, participants who reported being interested in both chocolate- and vanilla-flavored e-liquids received a total score of “1” for the other sweets flavor category.

Fourth, individual factors related to vaping were assessed (see Supplementary file 1 [38] for the items that were used). Evidence-based knowledge about smoking and vaping was measured with 12 statements that were either correct or incorrect. We consider evidence-based knowledge as knowledge that is based on scientific consensus—that is, information provided by the Dutch National Institute of Public Health and the Environment (RIVM) and previous research [19,48,49]. The knowledge items were summed (1 = correct, 0 = incorrect; ‘I don’t know’ was categorized as incorrect). Furthermore, a 7-point Likert scale was used to assess trust in information (two items) [50], perceived susceptibility towards e-cigarettes (three items: item A, item B, item C) [51,52], severity related to vaping (four items [51,52]), attitude towards e-cigarettes (four items) [39,48], social influence (one item) [53], deliberation about vaping (three items) [48], and intention to start using e-cigarettes (one item). A scale was computed for trust in information, severity related to vaping, attitude towards e-cigarettes, and deliberation of the pros and cons of vaping, by averaging the scores of the two items for trust (Cronbach’s α = 0.915), the four items for severity (Cronbach’s α = 0.639), the four items of attitude (Cronbach’s α = 0.927), and the three items for deliberation (Cronbach’s α = 0.656). No scale could be computed for perceived susceptibility towards e-cigarettes (Cronbach’s α ≤ 0.6)—thus, for each user group, the three mean scores for perceived susceptibility towards e-cigarettes and the three mean scores for perceived susceptibility towards cigarettes (for each individual survey item) were used. Similarly, for each user group, the mean score for each item regarding intention and social influence was determined.

### 2.3. Data Analysis

IBM statistics SPSS version 24 (IBM, Armonk, NY, USA) [54] was used for data analysis. No data were excluded. Attractiveness of product characteristics, and the e-liquid flavor categories preferred (for never-users and smokers, excluding those who selected the answer option “I don’t want to try any flavor”) and firstly and currently used (for dual and exclusive vapers) were analyzed using frequencies. Flavor preferences were presented in a pie chart as the percentage of the total number of responses.

Spearman correlation analyses showed that age (*p* < 0.05), gender, and level of education (<0.05) were significantly associated with individual factors related to vaping. However, these Spearman correlations were small, ranging from −0.211 to 0.169. Age, level of education, and gender were therefore excluded from further analyses due to small or non-significant correlations.

Individual factors were compared between both never-users and smokers interested in trying an e-liquid flavor and those not interested in trying a flavor (answer option: “none of the flavors”) using *t*-tests. Results were considered significant if *p* < 0.05.

## 3. Results

Of the 728 never-users, smokers, dual users, and exclusive e-cigarette users, 23.7% was highly educated (50.0% low education level and 26.2% middle education level), and the average age was 34.1 (± 20.2, min = 13, max = 84) (see Table 1).

### 3.1. Attractiveness of Product Characteristics

Table 1 shows attractiveness of e-cigarette product characteristics, stratified by user group. From the e-cigarette product characteristics assessed, all groups reported flavors as the most attractive.

### 3.2. E-Liquid Flavor Preferences

Of the 407 never-users, 68% selected the option “none of the flavors” (*n* = 278 participants) and 32% selected to be interested in trying one or more e-liquid flavor” and 32% (*n* = 248 responses)). Of the 138 smokers, 20% (*n* = 27 participants) were interested in none of the flavors and 80% (*n* = 208 responses) selected to be interested in trying one or more e-liquid flavor categories.

**Figure 1:**
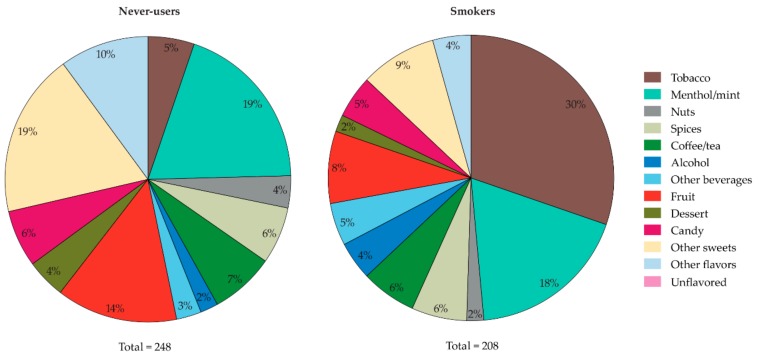
Interest in trying e-liquid flavors among never-users (left) and smokers (right). Never-users (*n*= 278; 68%) and smokers (*n* = 27; 20%) who selected the option “none of the flavors” were excluded from this visualization, and hence the pie charts visualize 248 responses from 32% of the never-users and 208 responses from 80% of the smokers. Data are presented as percentages of the total number of responses, not of the total sample sizes.

Figure 1 shows e-liquid flavor preferences as the percentage of each flavor category for never-users and smokers. Never-users were mostly interested in trying e-liquid flavors from the menthol/mint (19% of 248 responses) and sweet categories, such as other sweets (19%) and fruit (14%). Smokers were mostly interested in e-liquids with tobacco flavor (30%), followed by menthol/mint (18%) and other sweets (9%).

**Figure 2:**
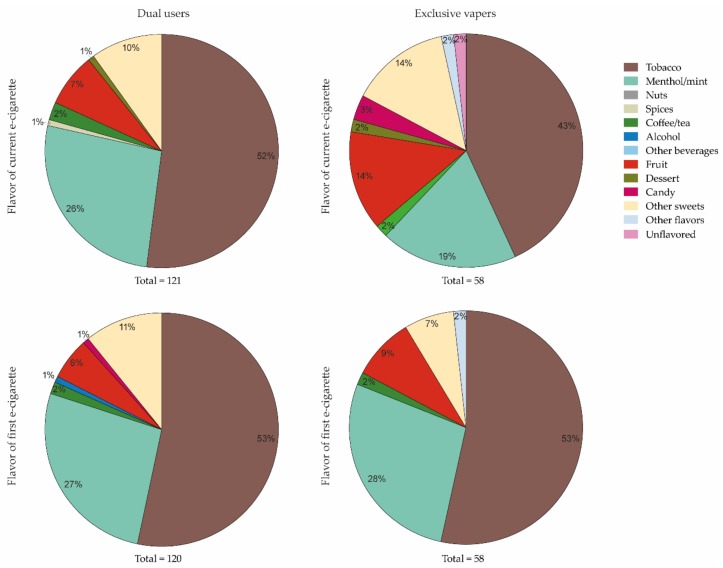
Flavors used on current (top) and first (bottom) e-cigarette exposure among dual (left) and exclusive vapers (right). Participants could select only one flavor category to indicate the flavor of their current and first e-cigarette used. Data are presented as percentages of the total number of responses, not of the total sample sizes.

Of the 122 dual users, 120 reported the flavor of their first e-cigarette used and 121 reported the flavor they currently use (see Figure 2). Of the 61 exclusive vapers, 58 reported the flavor of both their first and current e-cigarette. Among dual users, the most frequently reported flavors of their current and first e-cigarette used were similar: tobacco (52% vs. 53%), menthol/mint (26% vs. 27%), other sweets (10% vs. 11%), and fruit (7% vs. 6%). Among exclusive vapers, differences were observed in the most frequently reported flavors of their current and first e-cigarette used: tobacco (43% vs. 53%), menthol/mint (19% vs. 28%)), and fruit (14% vs. 9%) and other sweets (14% vs. 7%).

### 3.3. Individual Factors Related to Vaping

Table 2 shows differences in individual factors related to vaping between never-users and smokers. In addition, differences within the groups of never-users and smokers between those who were interested in trying an e-liquid flavor and those who did not want to try any flavor are shown. Never-users who were interested in trying a flavor had significantly less knowledge about e-cigarettes compared to never-users who did not want to try any e-liquid flavor (*p* < 0.05). Not surprisingly, never-users and smokers who were interested in trying a flavor were significantly more positive towards e-cigarettes and had a significant higher intention to start vaping, compared to never users and smokers who reported not wanting to try any e-liquid flavor (*p* < 0.05), within both never-users and smokers. Never-users who were interested in trying a flavor reported a lower perceived susceptibility (item C) than never-users who did not want to try a flavor (*p* < 0.05). In addition, smokers who were interested in trying a flavor considered the social influence towards not using e-cigarettes as less strong, which means that the smokers who were not interested in trying an e-cigarette flavor more often find that society thinks that one should not vape (*p* < 0.05).

## 4. Discussion

This study shows that the availability of different flavors was reported to be the most attractive product characteristic of e-cigarettes by all user groups, and that flavor preferences differ between never-users, smokers, dual users, and exclusive vapers. The first e-cigarette used by dual users and vapers mostly had a tobacco or menthol/mint flavor, but compared to dual users, we observed that exclusive vapers use more sweet- and fruit-flavored e-liquids than dual users. While tobacco was the most appealing flavor category among smokers, never-users were mostly interested in trying menthol- and sweet-flavored e-liquids. In addition, individual factors related to vaping differed within the groups of never-users and smokers. That is, never-users interested in trying a flavor had less knowledge about cigarettes and e-cigarettes than those who did not want to try any flavor. Attitude was more positive, and intention to start vaping was higher among both never-users and smokers who were interested in trying a flavor compared to those not interested in trying a flavor. Perceived susceptibility of health consequences was lower among never-users who were interested in trying a flavor, and social influence regarding not using e-cigarettes was lower among smokers who were interested in trying a flavor. Thus, similarly to the role of flavors in food choice [31,32], our results indicate that interest in flavors may moderate the differences in individual factors related to vaping.

While concerns have been raised about potential e-cigarette use among never-users [3,9,55], the never-users in our study had a low intention to start vaping and more than two-third (68%) of the never-users did not want to try any e-liquid flavor. However, nearly one-third of the never-users were still interested in trying an e-liquid flavor. Not surprisingly, they perceived a lower susceptibility towards negative health consequences of vaping, had a more positive attitude towards e-cigarettes, less knowledge about cigarettes and e-cigarettes, and a higher intention to start vaping than never-users who did not want to try any flavor. It should, however, be noted that a causal relation between these findings was not examined. For example, never-users could report to find e-liquid flavors interesting because they were already interested in trying e-cigarettes. On the other hand, they may have become interested in trying e-cigarettes because of the appealing flavors that they recognize from palatable food products. This means that being interested in flavors has a positive effect on the decision to start using e-cigarettes, or vice versa. Nevertheless, our findings regarding the interest of never-users in e-liquid flavors indicate that never-users may be vulnerable to flavor marketing of e-cigarettes [26,27,56,57]. For example, marketing of appealing e-liquid flavors may make never-users even more positive towards vaping, thereby potentially influencing their choice to initiate or refrain from vaping [19]. This suggests that the reverse can also be true: adapting product characteristics, for example restricting e-liquid flavors or regulating other product characteristics, may reduce attractiveness and consequently make never-users more negative about vaping. Some characteristics of e-liquids are currently regulated by Tobacco Regulation in the Netherlands [58,59]. However, legislation regarding e-liquid flavors currently does not exist. Further research is needed to help regulators decide whether and how the regulation of e-liquid flavors can improve public health.

This study showed that smokers who were interested in trying an e-liquid flavor had a more positive attitude towards e-cigarettes than smokers who were not interested in trying a flavor. In addition, smokers who were interested in trying a flavor considered the social influence towards not using e-cigarettes as less strong, which means that the smokers who were not interested in trying an e-cigarette flavor more often find that society thinks that one should not vape. Furthermore, two-third of the smokers reported interest in an e-liquid flavor other than tobacco. This indicates that flavors could support the decision of smokers to switch to vaping [57], for example by allowing the marketing of e-liquid flavors and other product characteristics that smokers find attractive [4]. The role of e-liquid flavors in supporting both the decision to switch towards e-cigarette use (for smokers) and to refrain from using e-cigarettes (for never-users) demonstrates the complexity of developing future regulations on e-liquid flavors.

Additional support for the interest in flavors moderating differences in individual factors related to vaping is provided by the different patterns of e-liquid flavors used by dual users and exclusive vapers. In line with previous research, both groups mostly used tobacco and mint flavored e-cigarettes at initiation, but exclusive vapers currently used more fruit and sweet e-liquid flavors than dual users [5,34,60,61,62]. This could be interpreted as vapers switching from tobacco to non-tobacco flavors over time, which is supported by a previous study [5]. Because most adult exclusive vapers included in this study used e-cigarettes for 1 to 5 years and most dual users reported to vape for only less than 6 months (data not shown), it is possible that the dual users may switch to fruit or sweet e-liquid flavors in the future. Longitudinal research is needed to investigate whether and how e-liquid flavors could support dual users in their decision to switch to exclusive vaping. In addition, it would be interesting to investigate the process of e-liquid flavors (or other product characteristics) eventually not living up to dual users’ expectation, thereby leaving them to quit vaping and relapse into exclusive cigarette smoking. This information could be used to, for example, stimulate an exchange of knowledge and experiences between exclusive vapers and dual users about the flavored e-liquids they use [63].

### 4.1. Future Research

Previous studies assessing individual factors related to vaping mostly focused their survey items on e-cigarettes in general. This means that participants are typically asked about their mental representation or beliefs of an unspecified e-cigarette, thereby not taking into account that the e-cigarette is a product that is available in various shapes, sizes, colors, flavors, and more. As our results suggest that flavors may moderate the differences in individual factors related to vaping, we recommend using survey items that represent a specific flavor or other product characteristic. For example, instead of only focusing on perceived susceptibility attitude towards e-cigarettes in general [64,65], researchers should also assess perceived susceptibility attitudes towards a specific e-liquid flavor categories, such as fruit, candy, and tobacco [47].

In addition, as other product characteristics may moderate differences found in individual factors related to vaping, the impact of for example price, labeling, and packaging of e-cigarettes and e-liquids on individual factors related to vaping should be investigated in different user groups [66] to determine which characteristics make up their “ideal” e-cigarette.

Furthermore, it would be interesting to use sensory research to investigate differences in e-liquid flavor liking between user groups, and how this relates to individual factors related to vaping. Finally, research is needed to investigate the interaction between product characteristics and individual factors related to new and emerging products, such as heated tobacco products and products containing nicotine salts. This will provide insight into which specific product characteristics are most appealing to vulnerable user groups, such as never-users and youth, and thus need to be regulated.

### 4.2. Limitations

Ideally, our sample size would be large enough to stratify our sample into different age groups and different flavor categories. However, our sample size was too small to determine differences in the preference of specific flavor categories between age groups (adults vs. adolescents), and differences in individual factors related vaping between specific flavor categories. In addition, response rates among adolescents was very low, and the rather high education level of participants in this study was not be representative of the Dutch population [67,68]. In addition, the sample cannot be considered representative of the Dutch population, as oversampling for the smokers and e-cigarette users was performed in order to have sufficient observations. As a result, the percentages of smokers and vapers in our study do not reflect the actual percentages of smokers and vapers in the Dutch population.

## 5. Conclusions

This study demonstrates that being interested in flavors moderates the differences in individual factors related to vaping for never-users and smokers, or vice versa. While the availability of different flavors was reported to be the most attractive product characteristic of e-cigarettes in all user groups, the specific flavor preferences varied between never-users, smokers, dual users, and exclusive vapers. Importantly, individual factors related to vaping (knowledge, perceived susceptibility attitude, social influence, and intention to start vaping) differed between never-users and/or smokers who were interested in trying an e-liquid flavor and those who did not want to try a flavor. Our results confirm the importance and complexity of regulating e-liquid flavors in a way that both the decision to switch towards vaping (for smokers) and the decision to refrain from vaping (for never-users) are supported. Ideally, regulation should allow marketing of e-liquid flavors that stimulate smokers and dual users to keep or start using e-cigarettes. To make never-users more negative about and keep them from using e-cigarettes, product appeal should be reduced by, for example, restricting the marketing and promotion of e-liquid flavors that they find particularly appealing.

## Figures and Tables

**Table 1:** Participants’ demographics and the attractiveness of e-cigarette product characteristics. Data are presented for adult and adolescent never-users (*n* = 407), smokers (*n* = 138), dual users (*n* = 122), and exclusive vapers (*n* = 61).

Participants’ Demographics and the Attractiveness of E-Cigarette Product Characteristics		Never-users (*n* = 407)	Smokers (*n* = 138)	Dual Users (*n* = 122)	Exclusive Vapers (*n* = 61)
Mean age (±SD)		31 (18.6) ^D^	35 (20.6)	37 (18.8) ^N^	37 (19.4)
Gender	Male	44.0%	37.7%	53.3%	49.2%
	Female	56.0%	62.3%	46.7%	50.8%
Education	Low	52.1%	52.9%	43.4%	39.3%
	Middle	20.6% ^D,E^	30.4%	35.2% ^N^	44.3% ^N^
	High	27.3%	16.7%	21.3%	16.4%
Attractive characteristics of e-cigarettes (%)	All the different flavors	10.3%	30.4%	34.4%	68.9%
	The product looks nice	6.6%	19.6%	22.1%	44.3%
	The nicotine level can be varied	4.7%	13.8%	15.6%	31.1%
	It is possible to alter the setting of the e-cigarette to my wishes	3.7%	10.9%	12.3%	24.6%
	Its varying designs	3.2%	9.4%	10.7%	21.3%
	You can blow nice smoke clouds with it (cloud chasing)	2.5%	7.2%	8.2%	16.4%
	Price of the product	2.0%	5.8%	6.6%	13.1%
	Price of the e-liquids	2.0%	5.8%	6.6%	13.1%

^N,D,E^ Superscripts indicate significant differences in a row between user groups (*p* < 0.05), with N = never-users, D = dual users, and E = exclusive vapers. Significant differences between user groups were determined for age, gender, and education using Bonferroni post-hoc corrections. General note: due to rounding, percentages may not add up to 100%.

**Table 2:** Individual factors related to vaping. Data are presented for never-users and smokers.

Individual Factors Related to Vaping			Never-users (*n* = 407)	Smokers (*n* = 138)
**Knowledge about e-cigarettes and cigarettes (±SD)**		Overall	9.3 (1.5) *	8.4 (1.8) *
		Those interested in trying a flavor	8.9 (1.7) °	8.4 (1.7)
		Those who did not want to try any flavor	9.4 (1.4) °	8.3 (2.1)
**Trust in information (±SD)**		Overall	5.2 (1.1)	4.9 (1.4)
		Those interested in trying a flavor	5.2 (1.0)	4.9 (1.4)
		Those who did not want to try any flavor	5.2 (1.4)	5.1 (1.5)
**Perceived susceptibility about vaping (±SD)**	A	Overall	4.9 (1.3) *	4.3 (1.2) *
		Those interested in trying a flavor	4.8 (1.3)	4.2 (1.2)
		Those who did not want to try any flavor	4.9 (1.3)	4.4 (1.2)
	B	Overall	5.0 (1.2) *	4.3 (1.2) *
		Those interested in trying a flavor	4.8 (1.2)	4.3 (1.2)
		Those who did not want to try any flavor	5.1 (1.2)	4.5 (1.2)
	C	Overall	4.9 (1.2) *	4.3 (1.2) *
		Those interested in trying a flavor	4.8 (1.3) °	4.2 (1.2)
		Those who did not want to try any flavor	5.0 (1.2) °	4.6 (1.2)
**Severity of vaping (±SD)**		Overall	4.6 (1.1) *	4.4 (1.1) *
		Those interested in trying a flavor	4.6 (1.1)	4.4 (1.2)
		Those who did not want to try any flavor	4.6 (1.1)	4.1 (1.1)
**Attitude towards e-cigarettes (±SD)**		Overall	2.1 (1.1) *	3.5 (1.1) *
		Those interested in trying a flavor	2.6 (1.2) °	3.7 (1.0) °
		Those who did not want to try any flavor	1.9 (1.0) °	2.9 (1.2) °
**Social influence (±SD)**		Overall	5.1 (1.7) *	4.4 (1.5) ^N^
		Those interested in trying a flavor	4.9 (1.7)	4.2 (1.5) °
		Those who did not want to try any flavor	5.2 (1.7)	5.1 (1.2) °
**Deliberation on the pros and cons of e-cigarette use (±SD)**		Overall	2.8 (1.5)	3.0 (1.5)
		Those interested in trying a flavor	2.9 (1.6)	3.1 (1.4)
		Those who did not want to try any flavor	2.7 (1.5)	2.7 (1.7)
**Intention to start vaping (±SD)**		Overall	1.2 (±0.8) *	2.5 (±1.7) *
		Those interested in trying a flavor	1.4 (1.1) °	2.7 (1.7) °
		Those who did not want to try any flavor	1.1 (0.6) °	1.6 (1.4) °

* Indicates significant differences (*p* < 0.05) in a row between user groups. ° Indicates a within-group significant difference (*p* < 0.05) between those who were interested in trying any e-liquid flavor and those who were not interested in trying any e-liquid flavor (only for never-users and smokers). Knowledge was determined using 12 statements. A higher score represents more knowledge, with 0 = no correct answers and 12 = correct answers for all statements. Trust was assessed with two items, using a 7-point Likert scale; 1 low to 7 = high level of trust in information provided. Perceived susceptibility assessed the chance of developing cancer as a result of vaping with three statements: (A) If I vape, then my risk of developing some form of cancer during my lifetime is…; (B) I think that if I vape, my risk of developing some form of cancer during my lifetime is …; (C) My feeling is that if I vape, the risk of developing some form of cancer during my lifetime is…; 1 = low to 7 = perception of cognitive risk of health risks related to e-cigarette use. Severity was assessed with four items, on a 7-point Likert scale ranging from very bad to not bad at all (1–7); Attitude was assessed with four items, using a 7-point Likert scale, with 1 = very negative towards e-cigarette use and 7 = very positive towards e-cigarette use. Deliberation was assessed with three items on a 7-point Likert scale, with 1 = no deliberation about E-cigarette use to 7 = very extensive deliberation about E-cigarette use. Intention to start vaping was reported on a 7-point Likert scale, with 1= very low intention to start vaping and 7 = very high intention to start vaping.

## Supplementary Materials

The following are available online at https://www.mdpi.com/1660-4601/16/23/4661/s1, Supplementary file 1 was previously described in Romijnders, Pennings, van Osch, de Vries and Talhout [38].
