# Supplementary material for: E-Liquid Flavor Preferences and Individual Factors Related to Vaping: A Survey among Dutch Never-Users, Smokers, Dual Users, and Exclusive Vapers
Source: Int J Environ Res Public Health. 2019 Nov 22;16(23):4661. doi: 10.3390/ijerph16234661 (PMC6926905; doi:10.3390/ijerph16234661)
Supplement: Supplementary file 1 [file ijerph-16-04661-s001.pdf]

E-liquid flavor preferences and individual factors related to vaping: a survey among Dutch never-users, smokers, dual users, and exclusive vapers

Kim AGJ Romijnders[1,2*], Erna JZ Krüsemann[1,3], Sanne Boesveldt[3], Kees de Graaf[3], Hein de Vries[2] and Reinskje Talhout[1]

[1] Centre for Health Protection, National Institute for Public Health and the Environment (RIVM), Antonie van Leeuwenhoeklaan 9, 3721 MA Bilthoven, the Netherlands; kim.romijnders@rivm.nl (K.A.G.J.R.); erna.krusemann@rivm.nl (E.J.Z.K.); reinskje.talhout@rivm.nl (R.T.).

[2] Department of Health Promotion, School for Public Health and Primary Care (CAPHRI), Maastricht University, Universiteitssingel 40, 6229 ER Maastricht, the Netherlands; hein.devries@maastrichtuniversity.nl (H.d.V.).

[3] Division of Human Nutrition and Health, Wageningen University, Stippeneng 4, 6708 WE Wageningen, the Netherlands; sanne.boesveldt@wur.nl (S.B.); kees.degraaf@wur.nl (K.d.G.).

* Correspondence: kim.romijnders@rivm.nl; Tel.: +31-30-274-4512

Table of Contents

Appendix A 2

Table A1: Overview of the included measures to determine user group, demographics, and attractiveness of product characteristics 2

Table A2: Overview of the included measures regarding flavor preferences 4

Table A3: Overview of the included measures regarding individual factors related to vaping 6

References 11

Appendix A

Table A.1. Overview of the included measures to determine user group, demographics, and attractiveness of product characteristics.

Concept	Item	Answer option	Explanation of the concept	Reference
**Type of user**	A. I smoke or vape	1. Both cigarettes and E-cigarettes<br>2. Only cigarettes<br>3. Only E-cigarettes<br>4. I do not smoke or vape<br>5. I have smoked in the past<br>6. I have vaped in the past<br>I have smoked both cigarettes and vaped in the past	• Response options A.4 formed the group never-users.<br>• Response options A.2 & B.1 OR B.2 formed smokers.<br>• Response options A.1 & (B.1 OR B.2) & C1.OR C2) formed dual users.<br>• Response options A.3 & Cs1 OR C.2 formed E-cigarette users.	Pearson, Hitchman (1)<br>Amato, Boyle (2),<br>International Tobacco Control Policy Evaluation Project (3)
	B. How often do you smoke?	1. Every day<br>2. Not every day, but at least once a week<br>3. Not every week, but at least once a month<br>Less than monthly		
	C. How often do you vape?	1. Every day<br>2. Not every day, but at least once a week<br>3. Not every week, but at least once a month<br>4. Less than monthly<br>I have never vaped regularly, I only tried it once or twice.		
**Demographics**	How old are you?	Open question		Centraal Bureau voor Statistiek (CBS) (4)
	What is your gender?	Male-female		

	What is your level of education?	1. Did not finish school<br>2. Primary school to 8th grade<br>3. Some high school, did not graduate<br>4. High school graduate, diploma or the equivalent (for example: GED)<br>5. Some college credit, no degree<br>6. Trade/technical/vocational training<br>7. Associate degree<br>8. Bachelor's degree<br>9. Master's degree<br>10. Professional degree<br>11. Doctorate degree	Low level of education: answer options 1, 2, 3, 4<br><br>Middle level of education: answer options 5, 6<br><br>Higher level of education: answer options 7 - 11	
**Attractiveness of e-cigarettes**	*The e-cigarette/vaper is attractive because (check all that apply)*	The product looks nice<br>Due to all the different flavors<br>Because it is possible to alter the setting of the E-cigarette to my wishes<br>Due to its varying designs<br>Due to the price of the product<br>Due to the price of the E-liquids<br>Because the nicotine level can be varied<br>Because you can blow nice smoke clouds with it<br>Not applicable, I do not find the E-cigarette/vaper attractive	Single item	Romijnders, van Osch (5), Romijnders, van Osch (6)

Table A.2. Overview of the included measures regarding flavor preferences.

**Concept**	**Item**	**Answer options**	**Explanation and formation of the concept**	
**Flavor preference of never-users and smokers**	If you were to start using an e-cigarette, which flavor would you like to try? *(check all that apply)*	• Tobacco<br>• Menthol/mint<br>• Nuts<br>• Herbs, spices<br>• Coffee/tea<br>• Cocktails<br>• Alcohol, other<br>• Sodas<br>• Sweet, chocolate<br>• Sweet, vanilla<br>• Sweets, other<br>• Fruit<br>• Sweet, desserts<br>• Milk products<br>• Candy<br>• Floral<br>• Unflavored<br>• None of the flavors	For never-users and smokers: if participants selected 'None of the flavors', no other flavor category could be selected simultaneously.<br><br>These closed answer options were recoded to the thirteen main categories of the recently published e-liquid flavor wheel (7), with the exception of "I don't want to try a flavor". Recoding reported flavor preferences resulted in the following thirteen main categories: *tobacco* (survey item: tobacco), *menthol/mint* (survey item: menthol/mint), *nuts* (survey item: nuts), *spices* (survey items: herbs, spices), *coffee/tea* (survey items: coffee; tea), *alcohol* (survey items: alcohol, cocktail; alcohol, other), *other beverages* (survey items: soda; sweet, other), *fruit* (survey item: fruit), *dessert* (survey items: sweet, dessert; milk product), *other sweets*	Yingst, Veldheer (8)
**Flavor preference of dual users and e-cigarette users**	*Which e-liquid flavor did you try first?*	• Tobacco<br>• Menthol/mint<br>• Nuts<br>• Herbs, spices<br>• Coffee/tea<br>• Cocktails<br>• Alcohol, other<br>• Sodas<br>• Sweet, chocolate<br>• Sweet, vanilla		

		• Sweets, other<br>• Fruit<br>• Sweet, desserts<br>• Milk products<br>• Candy<br>• Floral<br>• Unflavored	(survey items: sweet, chocolate; sweet, vanilla), *candy* (survey items: sweet, candy), *other flav*ors (survey items: floral; other) and *unflavored* (survey item: unflavored). For example, if a never-user or smoker reported an interest in the survey items “sweet, candy” and “alcohol, cocktail”, their answers were recoded as a preference for the categories candy and alcohol, respectively. Open answers from dual and e-cigarette users were assessed by two authors (KR and EK) to support recoding of their closed answers in accordance with the categories of the e-liquid flavor wheel (7).
	Which e-liquid flavor do you currently use most?	• Tobacco<br>• Menthol/mint<br>• Nuts<br>• Herbs, spices<br>• Coffee/tea<br>• Cocktails<br>• Alcohol, other<br>• Sodas<br>• Sweet, chocolate<br>• Sweet, vanilla<br>• Sweets, other<br>• Fruit<br>• Sweet, desserts<br>• Milk products<br>• Candy<br>• Floral<br>• Unflavored	

Table A.3. Overview of the included measures regarding individual factors related to vaping.

Concept	Item	Answer option	Explanation of the scale	Formation of the concept	Cronbach's alpha (α)	Reference
**Knowledge about tobacco product use and e-cigarette use**	1. The E-cigarette/vaper is 95% less damaging than a cigarette.	0 = incorrect<br>1 = correct<br>NA = don't know	0 = no correct answers to 9 = all statements were answered correctly	- 0 = no correct answers to 12 = all statements were answered correctly.<br>- The scores were summed to come to a final score of possible correct answers out of 12.<br>- *I don't know* was categorized as incorrect.<br>- The coding of correct and incorrect answers was based on scientific consensus.<br>- The statements assessing knowledge were based on previously conducted a focus group study.	NA	Romijnders, van Osch (6)
	2. Only water vapor comes out of an E-cigarette/vaper.					
	3. E-cigarettes/vapers are the same thing.					
	4. E-cigarette use can lead to irritation and damage of the airways, palpitations and an increased risk of cancer.					
	5. The E-cigarette/vaper is a scientifically proved means of stopping smoking.					
	6. There are just as many harmful substances in the E-cigarette liquid as in a cigarette.					
	7. Smoking increase the risk of developing various diseases, including lung cancer and various other types of cancer, cardiovascular disease and COPD.					
	8. Additives (substances added to the tobacco in a cigarette) cannot make cigarette smoke any more addictive.					
	9. Smoking is the main cause of premature death.					
	10. It is always good to stop smoking, even for a short time.					
	11. Passive smoking is also damaging.					

	12. Getting enough exercise compensates for the risks to health from smoking.					
**Attitude towards e-cigarettes**	*I think vaping is…*		1= very negative towards e-cigarette use to 7 = very positive towards e-cigarette use.	The four items were summed and averaged to compute one score of the concept Attitude towards e-cigarettes.	*0.927*	Lehmann, de Melker (9)
		really bad - really good (1-7)				
		really harmful - really safe (1-7)				
		really gross - really nice (1-7)				
		really socially unacceptable behavior - really socially acceptable behavior (1-7)				
**Deliberation on the pros and cons of e-cigarette use**	*Please keep your own decision in mind, but have you considered the option of using e-cigarettes?*		1 = no deliberation about e-cigarette use to 7 = very extensive deliberation about e-cigarette use	The three items were summed and averaged to compute one score of the concept Deliberation on the pros and cons of e-cigarette use.	*0.* 656	Lehmann, de Melker (9)
	I have visualized how it would feel not to smoke and not to vape.	I have not visualized how I would feel - I have visualized how I would feel (1-7)				

	I have visualized how it would feel to smoke.	I have not considered the consequences - I have considered the consequences (1-7)				
	I have visualized how it would feel to vape.	no conscious list of the pros and cons - a very conscious list of pros and cons (1-7)				
**Perceived susceptibility about vaping (cognitive)**	A. If I vape, then my risk of developing some form of cancer during my lifetime is…	very small - very big (1-7)	1 = low to 7 = high perception of cognitive risk of susceptibility to health risks related to e-cigarette use	Answer options were recoded to make sure 7 is high and 1 is low.<br><br>Cognitive susceptibility and severity items on side-effects could not be summed [6] because Cronbach's alpha was below 0.6. A concept could not be created, so the individual items were used.	NA	Janssen, van Osch (10)<br><br>de Vries, van Osch (11)
	B. I think that if I vape, my risk of developing some form of cancer during my lifetime is…					
**Perceived susceptibility about vaping (affective)**	C. My feeling is that if I vape, the risk of developing some form of cancer during my lifetime is…	very small - very big (1-7)	1 = low to 7 = high affective risk perception for susceptibility to	Answer options were recoded to make sure 7 is high and 1 is low.	NA	Janssen, van Osch (10)<br><br>de Vries, van Osch (11)

			health risks related to e-cigarette use.			
**Severity of vaping**	A. How bad would you feel if you developed a form of cancer during your lifetime?	very bad - not bad at all (1-7)	1 = low to 7 = high perception of severity of health risks	Answer options were recoded to make sure 7 is high and 1 is low.<br><br>The four items were summed and averaged to compute one score of the concept Severity.	0.639	Janssen, van Osch (10)<br><br>de Vries, van Osch (11)
	B. If I should develop a form of cancer during my lifetime, then I would die of it.	completely agree - completely disagree (1-7)				
	C. If I should develop a form of cancer during my lifetime then this would influence my relationships (e.g. family friends or colleagues).					
	D. If I should develop a form of cancer during my lifetime, then I am afraid that I will die of it.					
**Trust in information**	I think RIVM is trustworthy.	completely agree - completely disagree (1-7)	1 low to 7 = high level of trust in information provided by the Dutch National Institute of Public Health and the	The two items were summed and averaged to compute one score of the concept Trust.	0.915	Siegrist, Earle (12)
	I think RIVM is independent.					

			Environment (RIVM).			
**Social influence**	Society thinks that you should not vape e-cigarettes.	completely agree - completely disagree (1-7)		Single item	NA	Montano and Kasprzyk (13)
**Intention to start vaping**	Please indicate on a scale from 1 to 7 your intent to start vaping in the next 6 months	I do not intent to start vaping - I intent to start vaping (1-7)	1 = low intention to start vaping to 7 = high intention to start vaping	Single item	NA	Montano and Kasprzyk (13)<br><br>Schoren, Hummel (14)

References

1. Pearson JL, Hitchman SC, Brose LS, Bauld L, Glasser AM, Villanti AC, et al. Recommended core items to assess e-cigarette use in population-based surveys. Tob Control. 2018;27(3):341-6.
2. Amato MS, Boyle RG, Levy D. How to define e-cigarette prevalence? Finding clues in the use frequency distribution. Tob Control. 2016;25(e1):e24-9.
3. International Tobacco Control Policy Evaluation Project. ITC Netherlands (NL11_Pw) Waterloo, Canada: International Tobacco Control (ITC) Policy Evaluation Project.; 2017 [Available from: https://www.itcproject.org/files/ITC_NL11_Replenishment_S_web_Eng_Dut.pdf.
4. Centraal Bureau voor Statistiek (CBS). International Standard Classification of Education (ISCED) [Dutch version]. Heerlen: Centraal Bureau voor Statistiek (CBS); 2018.
5. Romijnders K, van Osch L, de Vries H, Talhout R. Perceptions and Reasons Regarding E-Cigarette Use among Users and Non-Users: A Narrative Literature Review. Int J Environ Res Public Health. 2018;15(6).
6. Romijnders K, van Osch L, de Vries H, Talhout R. A Deliberate Choice? Exploring the Decision to Switch from Cigarettes to E-Cigarettes. Int J Environ Res Public Health. 2019;16(4).
7. Krusemann EJZ, Boesveldt S, de Graaf K, Talhout R. An E-liquid Flavor Wheel: A Shared Vocabulary based on Systematically Reviewing E-liquid Flavor Classifications in Literature. Nicotine Tob Res. 2018.
8. Yingst JM, Veldheer S, Hammett E, Hrabovsky S, Foulds J. A Method for Classifying User-Reported Electronic Cigarette Liquid Flavors. Nicotine Tob Res. 2017;19(11):1381-5.
9. Lehmann BA, de Melker HE, Timmermans DRM, Mollema L. Informed decision making in the context of childhood immunization. Patient Educ Couns. 2017;100(12):2339-45.
10. Janssen E, van Osch L, Lechner L, Candel M, de Vries H. Thinking versus feeling: differentiating between cognitive and affective components of perceived cancer risk. Psychol Health. 2012;27(7):767-83.
11. de Vries H, van Osch L, Eijmael K, Smerecnik C, Candel M. The role of risk perception in explaining parental sunscreen use. Psychol Health. 2012;27(11):1342-58.
12. Siegrist M, Earle TC, Gutscher H. Test of a trust and confidence model in the applied context of electromagnetic field (EMF) risks. Risk Anal. 2003;23(4):705-16.
13. Montano DE, Kasprzyk D. Theory of Reasoned Action, Theory of Planned Behavior, and the Integrated Behavioral Model. In: Glanz K, Rimer BK, Viswanath K, editors. Health Behavior and Health Education: Theory, Research and Practice. 4th ed. New Jersey: John Wiley & Sons; 2008. p. 67-96.
14. Schoren C, Hummel K, Vries H. Electronic cigarette use: comparing smokers, vapers, and dual users on characteristics and motivational factors. Tobacco Prevention & Cessation2017.
